# Cardiac CT for electrophysiological interventions

**DOI:** 10.1007/s10554-025-03397-8

**Published:** 2025-05-27

**Authors:** Kosmas Maragiannis, Dominik C. Benz, Ardan M. Saguner, Alexander Breitenstein, Jonathan Michel, Aju P. Pazhenkottil, Philipp A. Kaufmann, Ronny R. Buechel, Andreas A. Giannopoulos

**Affiliations:** 1 Department of Nuclear Medicine, Cardiac Imaging, University Hospital Zurich, Raemistrasse 100, Zurich, 8091 Switzerland; 2 Department of Cardiology, University Heart Center, University Hospital Zurich, Zurich, Switzerland; 3 Center for Translational and Experimental Cardiology (CTEC), Department of Cardiology, Zurich University Hospital, University of Zurich, Schlieren, 8952 Switzerland

**Keywords:** CCT, Electrophysiology, Atrial fibrilation, LAAO, AI

## Abstract

Cardiac computed tomography (CCT) holds an important role in the field of electrophysiology offering critical insights that enhance the management of arrhythmias through precise procedural planning and execution. It has furthermore established its role as a useful imaging modality in left atrial appendage closure procedures. This review discusses the current applications of CCT from pre-interventional assessment to post-interventional follow-up, emphasizing its utility in improving the safety and efficacy of electrophysiological and left atrial appendage occlusion interventions. It also explores the integration of CCT with advanced technologies such as electroanatomical mapping systems and the emergence of innovative imaging modalities, including three-dimensional cardiac computational modelling. CCT’s evolving capabilities suggest a promising future in electrophysiology and left atrial occlusion procedures when combined with further technological advancements, including artificial intelligence software.

## Introduction

Cardiac computed tomography (CCT), characterized by high isotropic spatial resolution and continually improving temporal resolution, is an established imaging modality for planning and guiding of cardiac interventional procedures [1]. Electrophysiological interventional procedures are employed for the treatment and prevention of a wide range of arrhythmic disorders. CCT is increasingly used in the context of these procedures, as it delivers essential anatomical insights that improve procedural outcomes, reduce procedural duration, and increase safety [2]. Moreover, CCT can be particularly useful in identifying complications associated with procedures and also allows for post-interventional patient monitoring [3, 4]. This article reviews the current applications, roles, limitations, and future potential of CCT, comparing it with other cardiac diagnostic tools in electrophysiological interventions (Table 1). These include ablation therapies for atrial fibrillation (AF) and ventricular tachycardia (VT), and the implantation and extraction of transvenous cardiac implantable electronic devices. Furthermore, the utilization of CCT in left atrial appendage occlusion (LAAO) for prevention of systemic thromboembolism in AF patients is highlighted.

**Table 1:** Summary of the current applications of cardiac CT in various electrophysiological procedures

Intervention		Procedural stages	
	Pre-procedural	Peri-procedural	Post-procedural
**Atrial fibrillation ablation**	• Exclusion of thrombus in LAA • Visualization of the anatomy and size of LA and PVs • Delineation of the anatomy between LA, PV, PNs and esophagus, minimizing the risk of esophageal and PN injury • Evaluation of IAS anatomy and anomalies, determining patient eligibility for the procedure • Evaluation of factors that increase the recurrence risk of post-ablative AF	• Integration of 3D cardiac CT images to EAM systems	• Diagnosis of PV stenosis, planning and assessment of balloon angioplasty/stent implantation procedure • Diagnosis of esophageal injury and formation of AEF
**Left atrial appendage occlusion**	• Exclusion of thrombus in LAA • Evaluation of ostium, length, structure and size of LAA, landing zone of device • Measurement of LA volume and size, assessment of left PV anatomy and relationship to LAA • Visualization of structures adjacent to LAA • Evaluation of IAS anatomy and anomalies, determining patient eligibility for the procedure	• Overlay or fusion imaging of CCT-derived data into angiographic images, providing real-time guidance during the procedure.	• Diagnosis of post-procedural complications including DRT, PDL, device erosion
**Ventricular tachycardia ablation**	• Exclusion of LV thrombus • Assessment for coronary artery disease • Assessment of myocardial scar and fatty infiltrations, localization of the VT-triggering substrate • Evaluation of factors that worsen ablation outcomes • Visualization of the thoracic anatomy in epicardial ablation procedures	• Integration of 3D heart CT models to EAM systems	
**Cardiac resynchronization therapy**	• Assessment of cardiac venous system		
**Device extraction**	• Evaluation of venous patency, venous occlusion, lead adhesions and lead perforation before lead extraction procedures		• Prevention of venous adhesion-associated postoperative complications

LAA, left atrial appendage; LA, left atrium; PV, pulmonic vein; PN, phrenic nerve; IAS, interatrial septum; AF, atrial fibrillation; 3D, three-dimension; CT, computed tomography; EAM, electroanatomical mapping; AEF, atrioesophageal fistula; CCT, cardiac computed tomography; DRT, device-related thrombus; PDL, peri-device leak; VT, ventricular tachycardia

### Atrial fibrillation

AF is the most common sustained supraventricular arrhythmia, with an increasing incidence and prevalence worldwide. The association of AF with a higher risk of thromboembolic disease and all-cause mortality underscores the need for effective treatment, while a rhythm control treatment strategy has shown to lower the risk of cardiovascular death, stroke, acute coronary syndrome and hospitalization for heart failure [5–9]. Catheter ablation has emerged as a cornerstone in managing AF, outperforming antiarrhythmic drugs in both paroxysmal and persistent forms in preventing the recurrence of AF and reducing the arrhythmia burden, while intervention early in the disease’s natural history is crucial for successful arrhythmia control [10–14]. Catheter ablation is an intervention characterized by the delivery of ablative energy, mainly in the form of radiofrequency, cryotherapy or electroporation, to arrhythmogenic myocardial substrate using electrode-equipped catheters. By disrupting the electrical signal conduction to the adjacent myocardium, electrical isolation of the arrhythmogenic foci is achieved [15, 16]. 

The CCT protocol for examining the left atrium (LA) and pulmonary veins (PV) prior to ablation or assessing peri- and post-procedural complications slightly differs from common coronary CT angiography protocols, due to the unique dynamics of contrast material in the LA and left atrial appendage (LAA), and the minimal influence of cardiac contraction on these structures, which reduces motion artifacts [17, 18]. The protocol includes arterial-phase imaging akin to coronary artery assessment, since contrast opacification in the LA and PV occurs slightly earlier than in the ascending aorta. A protocol commonly used includes breath-hold acquisition with prospective electrocardiographic (ECG)-gating at end-inspiration. A recommended contrast agent protocol, involves injecting 50 ml of contrast at a rate of 5 ml/s, followed by a 50 ml bolus of a 50:50 contrast and 0.9% saline mixture, and concluding with a 25 ml flush of 0.9% saline. This sequence enhances the opacification of the LA and coronary arteries through rapid contrast injection. The mixed contrast-saline bolus also improves the visualization of right atrium, facilitating detailed anatomical assessments of the interatrial septum (IAS). Alternatively, a bolus of 60-100 ml (BMI/BSA-adapted) of contrast followed by 50 ml flush of 0.9% saline can be used with a slower rate of 4 ml/s. Images are acquired in end-diastole when prospective scanning mode is used, or alternatively three seconds after peak enhancement in the ascending aorta when a retrospectively ECG-gated CT is used, with image reconstruction during systole, when the LA is most dilated. For optimizing data co-registration with the free-breathing electroanatomical mapping (EAM), acquisitions during expiratory breath-hold may be performed.

**Fig. 1:**
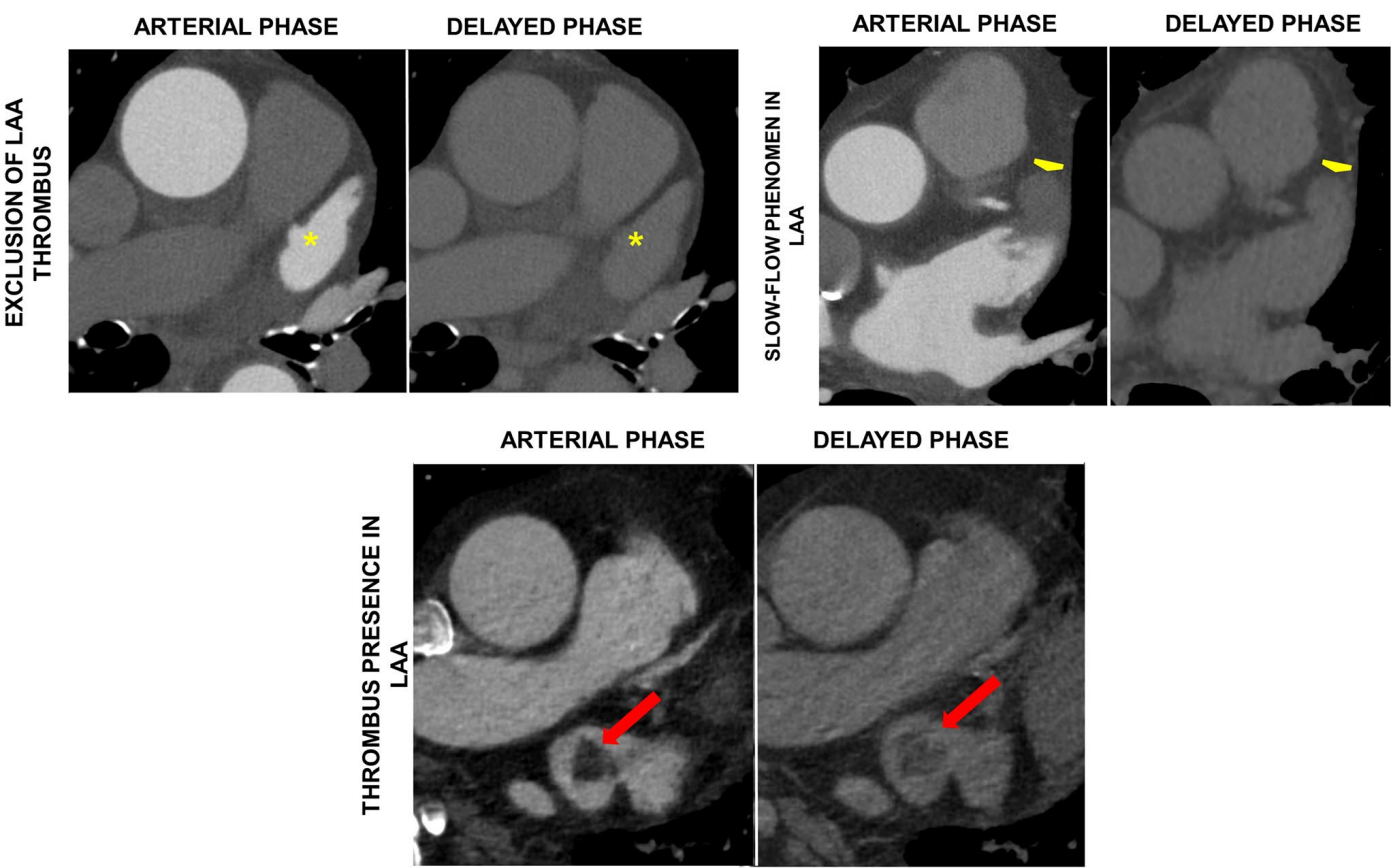
Assessment of LAA using CCT using arterial (left panels) and delayed phase imaging (right panels) in patients with atrial fibrillation planned for pulmonary vein isolation. Upper left panels depicts a representative example of a LAA free of thrombus, evident by the normal contrast enhancement in both phases. Upper right panels demonstrate an example of slow-flow phenomenon in a patient with severely dilated left atrium. Inhomogeneity and lack of contrast enhancement of the distal part of LAA in the arterial phase, which does not persist in the delayed imaging phase, where the LAA is homogenously contrasted. Lower panels depict an LAA thrombus (thrombus in transit) with a filling defect at the proximal part of the LAA in the arterial phase, which persists in the delayed imaging phase. LAA, left atrial appendage, CCT; cardiac computed tomography

Although transesophageal echocardiography (TEE) remains the gold standard for evaluating LAA thrombus, CCT can reliably exclude the presence of AF-associated LAA thrombi. At CCT, an LAA thrombus appears as a hypoattenuating contrast-filling defect, with homogeneous opacification indicating the absence of a thrombus, supported by a negative predictive value (NPV) of 98% (Fig. 1). However, contrast-filling defects may also result from the incomplete mixing of contrast material and blood during low flow states, explaining the low positive predictive value (PPV) of 20% for arterial-phase-only CCT scans in detecting LAA thrombus [19, 20]. To enhance diagnostic accuracy, various strategies have been developed. The most effective is the inclusion of a delayed imaging protocol, where a second set of images is acquired 30 to 180 s after the initial scan [21]. This method achieves a sensitivity of 100%, a specificity of 99%, a PPV of 92%, and a NPV of 100% in detecting LAA thrombi [22]. Other techniques include prone positioning during imaging, measuring iodine concentration using spectral CCT and employing specific contrast injection methods such as split bolus or double injection, followed by Hounsfield unit (HU) ratio calculation between the ascending aorta and LAA [23–25]. Commonly, a 60 to 90-second delayed-phase imaging is recommended to differentiate between a thrombus and slow blood flow, as alternative strategies lack supporting evidence and may disrupt workflow by requiring patient repositioning [21]. The additional acquisition of a second delayed scan (after about 120 s) may also be useful in equivocal cases.

**Fig. 2:**
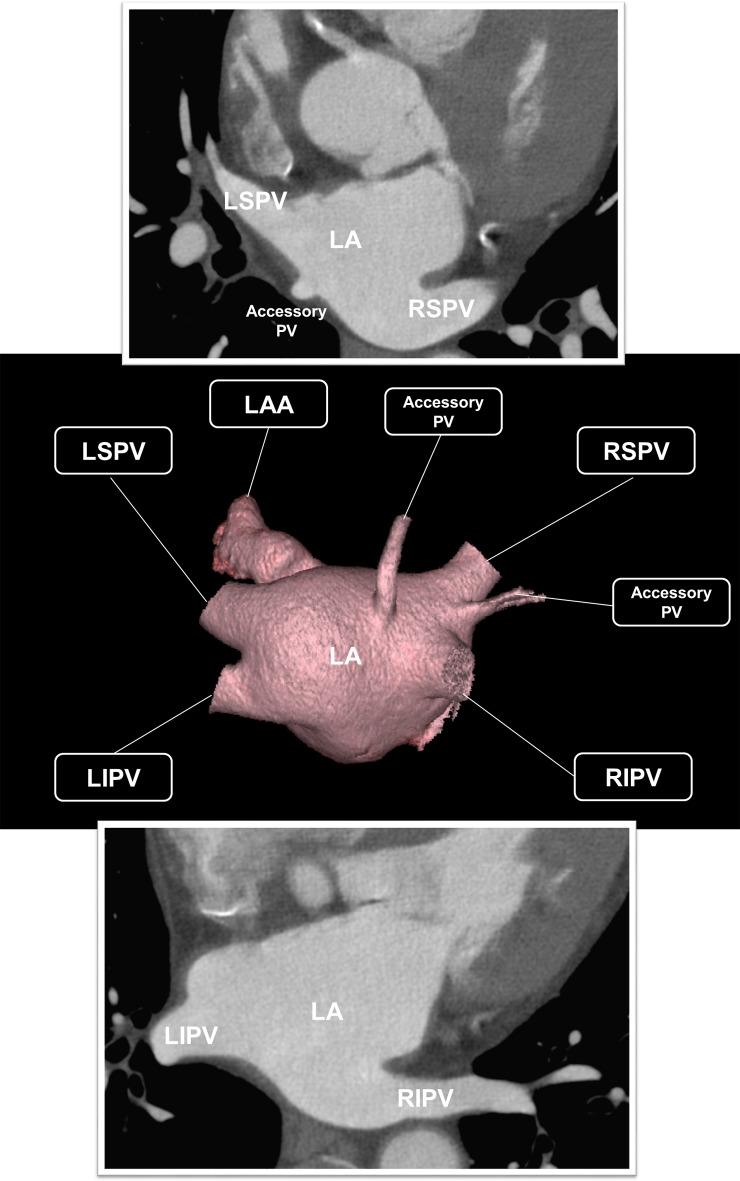
Anatomy of the LA and PVs at CCT using the axial images and 3D reconstruction. The most common configuration features four distinct PV ostia in the posterior LA on the right, the right superior and inferior PVs (RSPV and RIPV) have separate ostia, often divided by the LA wall, while on the left, the superior and inferior PVs (LSPV and LIPV) typically share closely positioned ostia without such separation. An accessory PV draining separately into the LA from the ipsilateral superior and inferior PV creates additional ostia. These accessory PV, often named based on the pulmonary segment they drain, are more commonly found on the right side than on the left, with an incidence reported in up to one-fourth of the population. When present, accessory PV trunks are typically shorter and narrower than the main PVs and may sometimes cross pulmonary fissures. LA; left atrium, PV; pulmonic vein, CCT; cardiac computed tomography, 3D; three-dimensional, LAA; left atrial appendage

The high spatial resolution of CCT enables the reliable evaluation of the anatomy and size of LA and PV and anatomic variations, including accessory PVs or common PV ostia (Fig. 2). CCT-derived morphology and dimensions of the PV and LA highly correlate with echocardiography and endocardial EAM findings, providing accurate anatomical data for planning ablation strategies [26]. CCT exhibits greater sensitivity than intracardiac echocardiography in identifying accessory PV branches and outperforms other modalities, including TEE, in determining the number of PV ostia, with CCT-derived PV ostial dimension measurements closely aligning with those obtained from intracardiac echocardiography and venography [27, 28]. Due to high-quality images, short-acquisition times and wide availability of post-processing software, CCT is often the preferred imaging modality for pre-procedural PV mapping [29]. By clearly delineating the anatomical relationships between LA, PVs and esophagus, CCT minimizes the risk of esophageal injury during ablation [30]. The personalization of ablation lines on the LA surface based on CCT data prior to the procedure, to avoid the area at risk of esophageal ablative damage, was associated with significantly reduced rates of esophageal injury, despite the moderate changes in esophageal position [31]. The close anatomical proximity of right and left phrenic nerve with the right superior PV and LAA respectively increases the ablation-induced injury of these structures [32]. Despite challenges in their visualization, CCT can be used to reconstruct the right and left pericardiophrenic bundles, indicating the location of the phrenic nerves, thus potentially identifying high risk patients and avoiding injury during ablation procedures [33–37]. 

CCT is valuable for assessing predictors of post-ablative AF recurrence. Factors such as anteroposterior LA diameter enlargement, common PV trunk, and presence of an accessory right-sided PV are considered independent risk factors for AF recurrence after radiofrequency catheter ablation [38]. Furthermore, CCT can quantify epicardial fat volume, thickness, and attenuation with high reproducibility [39, 40]. Although the relationship between the epicardial adipose tissue volume and AF recurrence remains ambiguous, increased attenuation and dispersion of epicardial adipose tissue—markers of heterogeneity—are associated with AF recurrence, independent of other clinical and echocardiographic factors [40–46]. Moreover, CCT can assess the LA wall thickness, a known risk factor in AF development and progression [47, 48]. LA wall thickness can be considered a surrogate marker in evaluating AF recurrence, as thickness of more than 3.10 mm might predict a poor response to ablation [49]. CCT-guided, LA wall thickness-based catheter ablation has been linked to reduced radiofrequency and fluoroscopy application and shorter procedural times [50]. Further studies are however required to confirm the role of CCT-measured atrial wall thickness on proper patient selection and catheter ablation approach tailoring [40]. 

Left-sided interventional procedures, including AF-ablation and LAAO, necessitate transseptal puncture (TSP) and CCT provides detailed information regarding the anatomy of the IAS, the presence of anomalies, including atrial septal defects, patent foramen ovale, septal aneurysms and lipomatous hypertrophy. CCT, combined with intra-operative TEE or intracardiac echocardiography can be used to guide transseptal procedures [51]. CCT-based three-dimensional (3D) reconstructions can be merged with real-time 3D EAM systems and utilized during ablation procedures. The process begins with segmentation, where structures of interest are delineated in CCT images and isolated from other structures. Following segmentation, co-registration integrates these images with EAM data, either by aligning landmarks identified during catheter mapping and visible in imaging or by matching the 3D anatomical endocardial shell of the mapped chamber with the image-derived contours. This integration of high-resolution anatomical details from CCT with electrophysiological data from EAM enables precise positioning of the ablation catheter relative to the LA and PVs. Nevertheless, while single-center observational studies and one randomized trial demonstrated superior efficacy and outcomes regarding long-term sinus rhythm maintenance with image integration, another randomized trial and meta-analysis found no significant benefit in rhythm outcomes [52–56]. 

CCT is central in diagnosing ablation-associated complications, such as pulmonary vein stenosis (PVS) and esophageal injury leading to atrioesophageal fistula (AEF). PVS can develop secondary to thermal injury-induced fibrosis and scarring of PV tissue and is associated with a significant morbidity [57, 58]. Although a prevalence up to 42% was reported with the earliest procedures, newer catheters and ablation techniques have decreased the incidence of severe PVS between 0.32% and 3.4% [57]. CCT with a PV enhancement protocol is the recommended method to anatomically assess PVS, providing precise description of PV anatomy and quantification of stenosis. PVS severity is categorized based on lumen diameter stenosis compared to a reference segment, as mild (< 50% lumen diameter stenosis), moderate (50–70%), or severe (> 70%) [59, 60]. Comparison of post-ablation CCT images acquired at the same point in the cardiac cycle as pre-ablation images is recommended due to physiological variations in ostial diameter [61]. Concomitant evaluation of the extracardiac organs can depict further PVS-specific changes in the pulmonic parenchyma and vasculature [39, 62]. Functional spectral perfusion mapping with chest CT serves as an alternative to ventilation-perfusion scintigraphy, categorizing the impact of PVS on lung perfusion. Moreover, CCT guides the planning of interventional PVS management including balloon angioplasty and stent implantation, providing essential anatomical details for fluoroscopic landmarks and angles [63]. Post-interventionally, serial CCT imaging could be considered for restenosis monitoring [63–67]. Although in some studies repeated CCT scans were performed as a routine screening for PVS following ablation, this practice is generally advised against due to the low incidence of severe stenosis [39, 57, 60, 68]. Serial CCT for asymptomatic PVS is also not recommended since treatment is typically unnecessary and PVS rarely progresses after the first three months post-ablation [69]. AEF, although rare, is a serious complication with high morbidity and mortality. CT is the preferred diagnostic method for AEF, distinguishing it from esophageal perforation and pericardioesophageal fistulas [10, 60, 70–73]. Diagnosis involves oral contrast administration to document esophageal perforation, typically indicated by contrast extravasation from the LA to the esophagus or air extravasation from the esophagus [73, 74]. 

Beyond ablation, CCT effectively excludes LAA thrombus in AF patients requiring electrical cardioversion [75]. Compared to TEE, CCT prior to cardioversion was associated with a shorter time-to-imaging, shorter time-to-cardioversion and improved Quality of Life of the patients at the time of hospital discharge [76]. Nevertheless, the use of CCT in AF patients has limitations, including radiation exposure, which can be further aggravated when additional delayed imaging is deemed necessary to exclude LAA thrombi. Additionally, the need for iodine-contrast agents may prevent it's use in patients with severe renal dysfunction or a history of anaphylactic reactions. Finally, in AF patients with very rapid ventricular response, the presence of motion artefacts might influence the diagnostic accuracy of the exam.

### Left atrial appendage occlusion

In AF patients with an estimated annual thromboembolic risk of ≥ 2%, oral anticoagulation is recommended to prevent stroke and systemic thromboembolism [10]. For moderate-to-high stroke risk patients with contraindications to long-term oral anticoagulation or high risk of major bleeding, LAAO can be considered an alternative management approach [10]. While TEE remains the gold standard for preprocedural LAA evaluation, CCT is also applied in pre-, intra- and post-interventional phase [21]. CCT reliably excludes the presence of LAA thrombus, a relative contraindication to LAAO, while providing high-resolution anatomical details, essential for procedural planning. These include the structure, size, and length of the LAA, its ostial dimensions and anatomical relationship to the left PV, the device landing zone, as well as anatomical characteristics of the IAS. Integration of CCT data with modern technologies including 3D computational modeling (3DCM) and 3D printing can potentially enhance the precision and outcomes of LAAO procedures. Post-operatively, CCT can be used for identifying LAAO-associated complications, including device-related thrombus, peri-device leak and device erosion.

For optimal visualization of LAA, the use of at least 64-slice CT scanner with ECG-gating is recommended to account for the dynamic size changes of the LAA throughout the cardiac cycle [77]. Adequate patient hydration is essential, and some centres advise pre-loading with 500 ml of NaCl prior to scanning to mimic the physiological volume status during implantation. Image acquisition should employ either prospective- or retrospective- ECG triggering, ideally during the ventricular systolic phase. Iodine contrast, commonly 80–100 ml at a flow rate of 4.5–5 ml/s is administered, followed by a 50 ml saline flush at the same rate. Images are captured from the carina to the diaphragm during expiratory breath-hold [21, 77]. Additionally, a delayed phase scan should be acquired 60 s after the arterial phase to assess for LAA/LA thrombus presence.

CCT effectively visualizes the LAA structure and size, where complex and variable anatomical morphology can influence procedural success. The LAA is often multilobed, featuring various lobe orientations and bends, and tapering towards its distal end. LAA morphology can be broadly classified into four shapes based on the number of lobes, bend angle, and presence of a dominant lobe [78]. Each shape correlates with different stroke risks and procedural difficulties [77]. The most commonly observed shape, the chicken-wing type, is present in approximately 48% of patients. It is characterized by a sharp bend angle (< 100º) in its proximal or middle portion or is folded back on itself [77]. This increases procedural complexity but is associated with the lowest incidence of stroke [17, 79]. A chicken-wing configuration with an upward bend, defined as a reversed chicken-wing, is anatomically associated with a lower position of LAA and a more proximal bending, explaining the increasing procedural complexity and significantly lower LAAO success rates compared to the other subtypes [80]. The risk of adverse cardiac and cerebrovascular events is also higher in reversed chicken wing subtypes [81]. The cactus type, accounting for 30% of cases, features a short, multilobed structure with a dominant, straight lobe, facilitating straightforward measurements and device implantation [77]. Seen in 19% of patients, the windsock type has a single dominant lobe, simplifying preprocedural measurements and device implantation. The least common shape, found in 3% of patients, is the cauliflower type, which is characterized by a short and irregular shape, without a dominant lobe and is linked to technically challenging device implantation, while associated with an eightfold increase in stroke risk compared to the chicken-wing type [77, 78]. 

CCT is instrumental in evaluating the ostium, length of the LAA, and the device landing zone—key factors for accurately sizing the LAAO device. The landing zone is defined as the segment within the LAA where the device must be placed so that its atrial end aligns properly with the LAA ostium, effectively occluding it. Two-dimensional (2D) oblique transverse measurements are preferred over 2D orthogonal or 3D measurements for their accuracy and reproducibility [82]. Compared to 2D-TEE or real-time 3D-TEE, CCT provides larger LAA dimensions but more accurate measurements of the LAA landing zone and ostial diameter, resulting into a more reliable device sizing [83–89]. Additionally, CCT provides anatomical information of the left PVs and their relation to the LAA ostium, crucial factors for procedural success, as in most transcatheter LAAO cases the left superior PV serves as the anchoring structure of the delivery catheter, further stabilizing the guidewire, across which the delivery sheath enters the LAA [77, 82]. Similarly, CCT allows for detailed appreciation of adjacent structures that are at risk of injury during the procedure, including the pulmonary trunk, left pulmonary artery, left circumflex artery, great cardiac vein, left phrenic nerve and Bachmann bundle (located lateral to ligament of Marshall, encircling the LAA neck) [77]. CCT can also assess anatomical variations or anomalies of the IAS, which might influence the TSP approach during LAAO procedures whereby coaxial alignment between the delivery sheath and LAA is desired. Furthermore, depending on the LAA type, different puncture strategies may be selected, for example a more central or anterior TSP is preferred for reverse chicken-wing LAA configuration or for a posterior-bending proximal LAA [90]. 

Advancements in CCT-based 3DCM allow for sophisticated 3D reconstruction of cardiac structures and simulation of procedures [91]. Specialized software platforms including, but not limited to, FEops HEARTguide™ (FEops nv, Gent, Belgium), 3mensio (Pie Medical Imaging, Maastricht, The Netherlands) and Mimics (Materialise, Leuven, Belgium), enable 3D assessments of the LAA and automatic, precise measurements from 3D models. This technology facilitates direct visualization of the TSP and device delivery as well as predetermination of fluoroscopic angles. Studies indicate that preprocedural planning with 3DCM is associated with more successful device implantations, improved procedural efficiency, reduced procedure times, fewer device resizing, and less device wastage [84, 88, 92]. Simulating procedural fluoroscopic coplanar views has been shown to allow for proper device deployment and reduced use of contrast material and radiation exposure [85]. Additionally, visualizing the projection and angulation between the LAA and TSP site aids in locating the optimal puncture site and selecting the appropriate delivery sheath shape [21]. Personalized LA and LAA 3D printed models further refine device selection and reduce the learning curve for procedural techniques, offering advantages over conventional CCT- or TEE-based methods [93–96]. Further, future applications include the fusion or overlay imaging of CCT-derived imaging onto real-time intraprocedural fluoroscopic angiograms, providing real-time guidance [21]. 

**Fig. 3:**
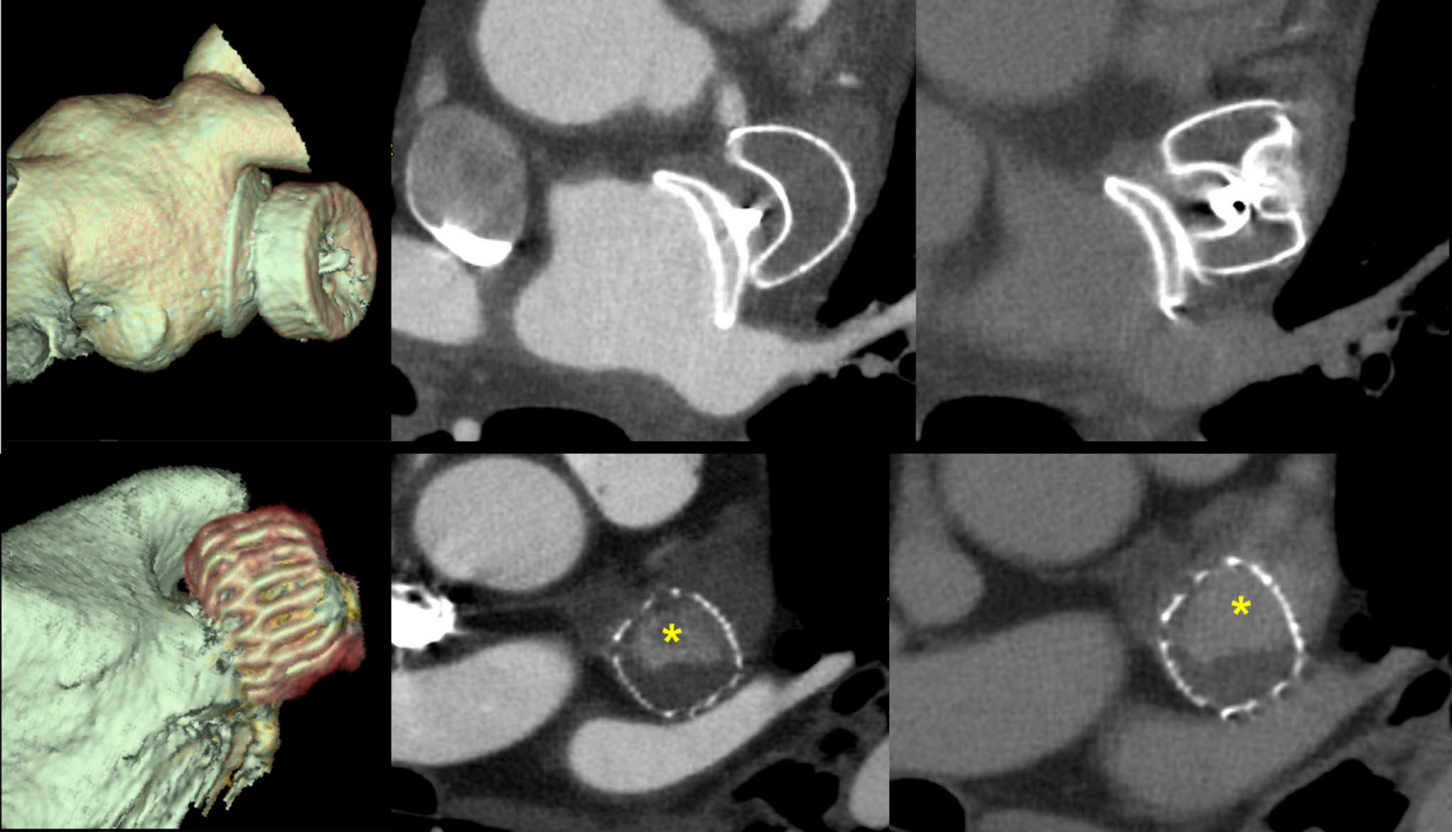
Post LAAO assessment using CCT. Upper panel depicts an Amplatzer Amulet 31 mm occluder device with completely thrombosed LAA, evident by the lack of perfusion in the arterial and delayed imaging phase (from left to right). Lower panel depicts a Watchman FLX 24 mm occluder device with rest perfusion (asterisk) of the only partially thrombosed LAA, in both the arterial and delayed imaging phase (from left to right). LAAO; left atrial appendage occlusion, LAA; left atrial appendage, CCT cardiac computed tomography

Post-procedurally, CCT is used for assessing implantation results and potential complications. Device-related thrombus, defined as thrombus formation on the atrial surface of the device, is identified on CCT as areas of hypoattenuated thickening [97] (Fig. 3). Peri-device leak, another complication following LAAO, manifests as a trail of contrast enhancement adjacent to the device and is further categorized by the extent of the leak: minimal (< 1 mm), mild (1–3 mm), moderate (4–5 mm), or severe (> 5 mm) [98, 99]. CCT assesses for residual patency in the LAA due to contrast leakage through the device, by measuring the linear attenuation coefficient within the LAA. An attenuation of < 100 HU and < 25% of the left atrium’s contrast opacification indicates successful LAA occlusion and absence of contrast leakage through the device [100]. Due to its high sensitivity, CCT is preferred over TEE for diagnosing peri-device leak [101]. CCT is also able to visualize fabric edge leak, defined as the residual leak occurring from a tilted device, not properly aligned to the axis of LAA. Following intradevice plugging, follow-up CCT was used to confirm complete leak closure, implying the feasibility and success of this novel treatment method for managing fabric edge leak [102]. Additionally, CCT assesses for incomplete neo-endothelialization of LAAO devices defined as LAA patency in the absence of peri-device leak [103, 104]. Lastly, while rare, device erosion through the LAA and pulmonary artery is a serious complication that can lead to cardiac tamponade and death, occurring weeks to months post-procedure. CCT aids in the diagnosis by visualizing the device’s location and the site of bleeding [105]. 

### Ventricular tachycardia

In patients with coronary artery disease, heart failure, or cardiomyopathies that experience VTs with implantable cardiac defibrillator interventions, VTs that are refractory to antiarrhythmic drugs, incessant VT, or electrical storm due to sustained monomorphic VTs, catheter ablation (endocardial or epicardial) is recommended as primary or secondary management strategy [106]. CCT is valuable in the pre-ablation phase for excluding left ventricular (LV) thrombus, identifying coronary artery disease as a potential cause of VT, pinpointing the anatomical substrate responsible for VT, assessing factors that may influence ablation outcomes, integrating anatomical information with EAM during the procedure and delineating thoracic anatomy for planning epicardial VT ablation procedures. Additionally, the development of stereotactic radio-ablation procedures employs CCT for precise planning.

Dedicated CCT protocols can allow for identification of myocardial scar presence and extent. Commonly, arterial-phase acquisition after administration of iodine contrast agent at doses of at least 1.5 ml/kg and up to 5 ml/kg, is followed by delayed scan acquisition at 90 s for exclusion of thrombus and at 7–10 min post-injection with lower tube current and voltage, in order to increase contrast-to-noise ratio [39, 107–110]. As the CT-derived biventricular volume and systolic function quantification correlate with good accuracy when compared to the gold-standard cardiac magnetic resonance (CMR), the entire cardiac cycle (0 to 100% of the R-R cycle) could be used for CCT acquisition [108, 111, 112]. Although this increases the radiation dose, the insights gained on biventricular function are of particular importance in evaluating the right ventricle, where identification of myocardial fibrosis is challenging [108]. Effective image post-processing is essential to optimize image quality and accurately identify myocardial fibrosis [108]. 

For endocardial ablation procedures, imaging is crucial to exclude the presence of LV thrombi. CCT and CMR detect LV thrombus more accurately than transthoracic echocardiography [39]. While CCT can effectively rule out LV thrombus, cine CMR and late gadolinium enhancement CMR are more commonly employed in clinical practice, particularly in patients without transvenous cardiac implantable electronic devices or in those with MR-safe electrodes, due to the limited data on CCT’s diagnostic accuracy and usage compared to other modalities for identifying LV thrombus [113, 114]. 

**Fig. 4:**
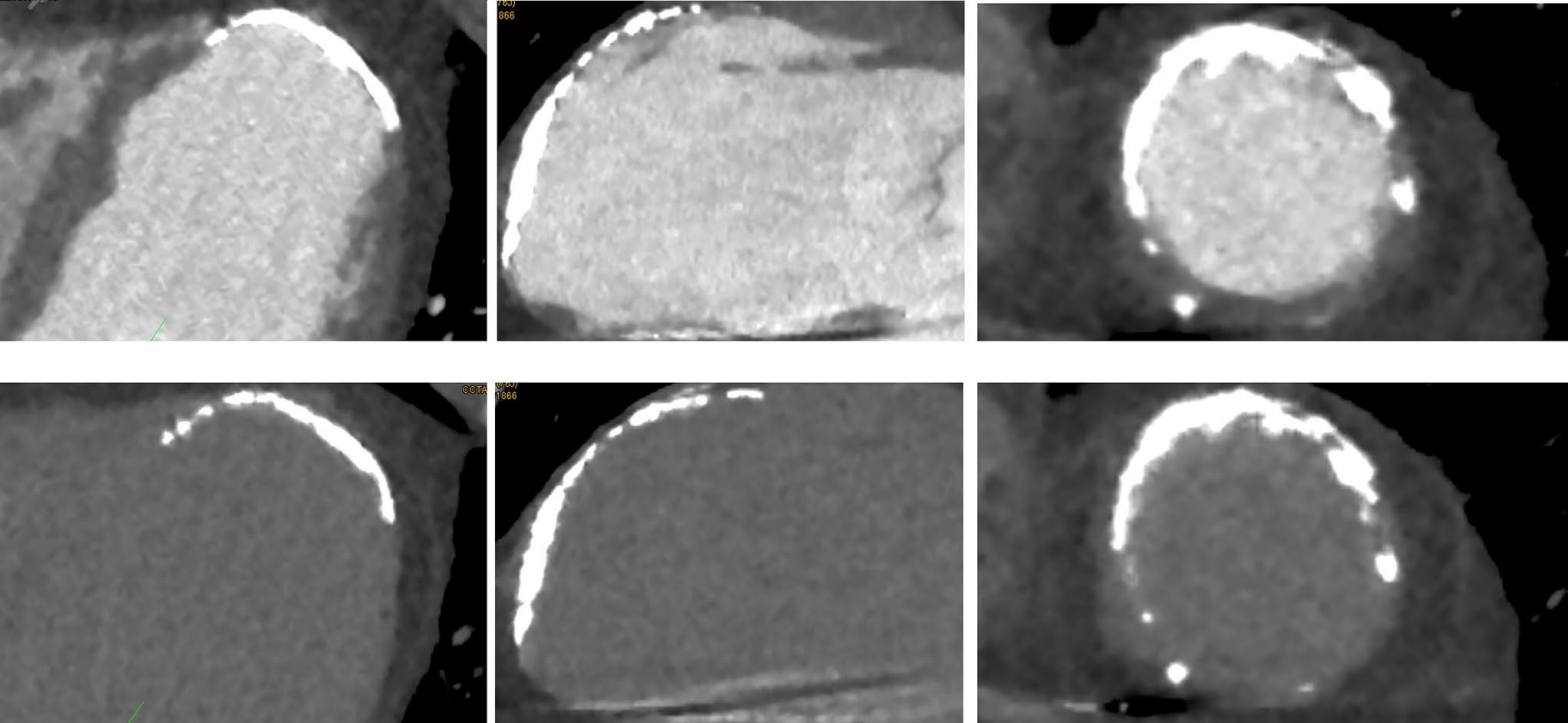
Use of CCT in the context of VT ablation. A 55-years old patient with ischemic cardiomyopathy and history of extensive myocardial infarction underwent a CCT for exclusion of LV thrombus in the LV. Upper panel demonstrates three chamber, two chamber and short axis CCT contrast enhanced images of the LV. Lower panel depicts the delayed phase imaging whereby a thrombus can be safely excluded based on the homogenous enhancement of the severely calcified, akinetic regions of the LV apex, the anterior and anteroseptal LV myocardium. The same contrast-enhanced CCT images were utilized peri-interventionally to guide the ablation procedure. LV; left ventricle, CCT; cardiac computed tomography, VT; ventricular tachycardia

Several studies have highlighted CCT’s capacity to detect myocardial scars, VT-triggering substrates, in patients with ischemic cardiomyopathy (ICM) undergoing VT ablation. Contrast-enhanced CT-derived data demonstrate high correlation with EAM for scar detection. Specifically, myocardial areas with wall thinning less than 5 mm at CCT are significantly associated with EAM-derived endocardial low-voltage areas (bipolar voltages less than 1.5 mV) and most ablation target sites correspond to CCT-identified VT channels of abnormal but a relatively thicker wall compared to surrounding tissues [115–120] (Fig. 4). Although CCT’s ability to characterize myocardial scars is considered less robust than CMR, advancements such as the integration of spectral CCT and late iodine enhancement (LIE) are promising, showing results comparable to late gadolinium enhancement-CMR [121–124]. Further research supports CCT’s use in measuring myocardial wall thickness and assessing myocardial viability, providing insights akin to those from CMR [107, 125]. Additionally, CCT is adept at identifying myocardial fat and calcifications, factors linked to VT ablation sites and poorer outcomes in ICM patients [126–129]. 

In non-ischemic cardiomyopathy (NICM), the efficacy of CCT for scar identification and characterization remains uncertain, with studies indicating a lack of a robust correlation between wall thickness under 5 mm and abnormal voltage areas [117, 119]. This inconsistency highlights the limited current potential of CCT in delineating scars that could serve as VT substrates in NICM, in part because fibrosis is often diffuse in NICM, necessitating further research to pinpoint specific scar characteristics and determine the optimal imaging modalities for this diverse patient group [39, 130]. However, LIE-CCT has been successful in visualizing NICM-associated myocardial scars and predicts low-voltage areas with higher accuracy. These findings support the potential of CCT in both preprocedural planning and intraprocedural scar localization, indicating its evolving feasibility in managing NICM [118, 131, 132]. 

CCT may be valuable in assessing patients with arrhythmogenic right ventricular cardiomyopathy (ARVC), where it identifies fatty infiltrations and tissue heterogeneity, parameters that correlate with low-voltage areas and VT-related sites [133, 134]. CCT reveals LV intramyocardial fat in the majority of ARVC patients [135]. Although these areas are associated with abnormal electrogram findings, voltage mapping may fail to detect them, highlighting CCT’s utility in localizing intramyocardial fatty infiltration and navigate electrophysiologists during the mapping phase of ARVC ablation procedures [136]. Furthermore, CCT-derived data showing intramyocardial fat can be integrated with EAM systems [39]. Through assessment of tissue heterogeneity, a surrogate for fibrofatty replacement, CCT successfully identifies VT substrates, as most abnormal electrograms and late potentials occur within fibro-fatty areas [134, 137]. 

CCT is instrumental in visualizing the entire thoracic anatomy, including large cardiac vessels and the pericardiophrenic bundles, contributing to complex epicardial ablation procedure planning [138]. By precisely identifying anatomical landmarks, CCT minimizes complications associated with epicardial VT ablation, such as injury to coronary arteries and phrenic nerves [139]. The accurate segmentation and integration of cardiac and extracardiac structures during ablation helps avoid coronary angiography and assists in phrenic nerve localization through high-output pacing [39]. Furthermore, the real-time integration of CCT-derived data during ablation procedures allows operators to focus high-resolution voltage mapping on myocardial scars, potentially harboring VT-triggering substrates, with studies reporting reduced procedural and fluoroscopic times. Although real-time imaging integration is linked to improved procedural outcomes compared to historical cohorts without imaging, the data on its efficacy are not uniformly consistent [140, 141]. 

Novel CCT technologies are expected to improve the efficiency and safety of VT ablation procedures. Traditionally evaluated by CMR, myocardial fibrosis can now be assessed in CCT with various protocols, in a feasible way and comparable to CMR, by determining the extracellular volume fraction [109, 110, 142–144]. This fraction indicates the percentage of extracellular matrix, thus fibrosis, within the myocardium. Extracellular volume estimation in CCT utilizes iodinated contrast to calculate the iodine concentration ratios between myocardium and blood-pool in late enhancement scans. This can be achieved either through single-energy subtraction of non-enhanced from late enhancement scans or by generating quantitative iodine maps from late enhancement spectral CT [110, 144]. Delayed imaging with photon-counting detector-CT, significantly improving spectral image quality and directly benefiting myocardial fibrosis assessment, allows for ECV calculation from a single late enhancement dual-source scan at a low radiation dose with high accuracy, eliminating the need for a second non-enhanced scan and thus avoiding potential misregistration issues [145, 146]. The quantification of extracellular volume by photon-counting detector-CT has also demonstrated good correlation with CMR, supporting its feasibility and future application in characterizing myocardial scars for patients where CMR is contraindicated [145, 147]. 

CCT is expected to advance the characterization of micro-architectural changes in the myocardial extracellular space, reflecting the biomechanical properties of the LV in response to various myocardial diseases. Texture analysis of LIE-CCT images has been effective in identifying myocardial heterogeneity, revealing distinct patterns of structural remodeling in patients with recurrent VT of diverse etiologies [148]. Moreover, recent advancements have enabled the integration of CCT-derived 3D heart models, generated from 3DCM, with EAM. CCT data can be uploaded to cloud-based platforms, whereby artificial intelligence-based segmentation algorithms perform post-processing analysis to generate detailed 3D heart models. Such advancements enhance the understanding of cardiac anatomy, streamline procedural workflows, improve outcomes, and reduce VT recurrence by optimizing ablation strategies [149]. 

CCT has been also utilized in the context of stereotactic radio-ablation procedures, a novel technique that applies stereotactic body radiotherapy (SBRT) for the ablation of VT [108]. Prior to SBRT, CCT assesses for coronary artery disease and myocardial scarring. Subsequently, a free-breathing simulation CT, encompassing the entire lung field, is performed to evaluate the patient’s thoracic anatomy and ensure proper positioning during the procedure. A 4-dimensional CT captures the thorax respiratory movements, providing crucial data for treatment planning. Electrophysiological data from EAM are integrated with simulation-CT to define the clinical target volume and the specific myocardial area targeted by SBRT. This technique offers a promising alternative for patients who have failed catheter ablation [150]. Early studies indicate that SBRT is effective and safe, significantly reducing the short-term VT recurrence, up to six months [151–154]. 

### Cardiac resynchronization therapy

Cardiac resynchronization, aiming to improve LV mechanics through biventricular pacing, is an established therapy in selected heart failure patients with reduced ejection fraction and electrical dyssynchrony, leading to reduced morbidity and mortality [155, 156]. In the pre-procedural setting, CCT contributes to guidance of proper placement of LV lead, through visual assessment of the cardiac venous system. The CCT protocol for imaging cardiac veins follows the standard CT angiography guidelines with a few important modifications. Optimal enhancement of the venous system is achieved with venous-phase imaging, where an 10–15 s delay is added to the contrast agent transit time [18]. Images are typically reconstructed during the systolic phase, when cardiac veins are most plethoric [157]. Assessing the coronary sinus, the presence of the Thebesian valve, and the cardiac veins that drain the posterolateral and lateral wall is crucial, as these anatomical features constitute the targets for LV lead implantation.

Despite the efficacy of CRT, up to 30% of patients do not respond to the therapy [158]. A crucial factor is the suboptimal placement of the LV lead; positioning it near the non-scarred LV wall with the most delayed mechanical activation significantly enhances response rates [159, 160]. The placement of the LV lead on the non-scarred and late-activated LV walls with the help of multimodal imaging, including CCT venography, significantly reduced CRT non-response rates [161]. Considering the contribution of CCT in assessing LV mechanical dyssynchrony and CRT outcomes, it’s role in CRT procedures has the potential to be further explored [161]. 

### Device extraction

Over the longer term, implanted transvenous devices can have serious complications, necessitating lead extraction due to infections or lead failure [162]. CCT visualizes venous patency, occlusions, and the lead’s trajectory within the venous system, providing insights in the preoperative planning. Its high spatial resolution is particularly effective in identifying lead adhesions and accurately depicting lead placement within heart chambers, crucial for evaluating potential lead perforation, which significantly influences the extraction planning. CCT-based holograms have the potential to further optimize the device extraction procedures.

**Fig. 5:**
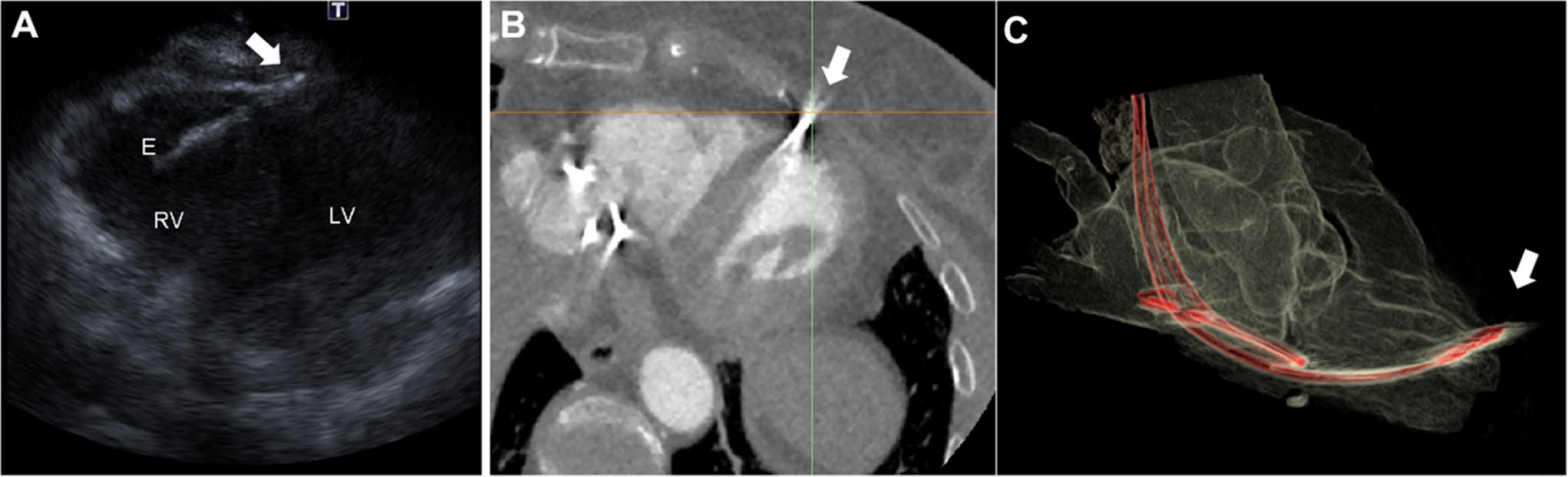
CCT for CIED assessment. A 63-years old woman with a history of dual chamber pacemaker implantation due to sick sinus syndrome, 10 years priorly and a revision one year ago due to pneumothorax, was presented with a few days history of diaphragm and thoracic muscle stimulation. Clinical examination and electrocardiogram were unremarkable. Transthoracic echocardiogram revealed a normal systolic function of the right and the left ventricle, yet raised the suspicion of presence of the right ventricle pacemaker electrode outside of the pericardium, with minimal fluid collection (Panel **A**). CCT further enhanced the suspicion of perforation of the right ventricular myocardium that was confirmed with three-dimensional reconstruction of the cardiac muscles, the pacemaker leads and the thoracic bone structures (Panel **B** and Panel **C**). The right ventricle electrode was completely removed and a new pacemaker implanted. CIED; cardiac implantable electronic device, CCT; cardiac computed tomography

CCT imaging prior to lead extraction is helpful in identifying patients with increased risk for complications by identifying intracardiac lead perforation (Fig. 5), a finding that impacts the preprocedural planning and the extraction process [163, 164]. Studies show that CCT outperforms CMR and 2D/3D echocardiography in accurately visualizing the position of the RV lead tip [165]. According to current expert consensus, CCT is the preferred modality for diagnosing lead perforations and influences the approach to lead extraction procedures by predicting potential challenges based on venous adhesions [162]. CCT-detected severe lead adhesions predispose to complex extraction procedures, major complications and confer worse outcomes, especially in patients with indwelling leads for less than ten years. This underscores the high clinical value of CCT in this patient group, where lead adhesions are not expected, as the presence of severe lead adhesions can influence the operative strategy and warrant further surgical precaution measures [166]. 

CCT holds a significant role in advancing new technologies that enhance the conductance and success rate of complex lead extraction procedures. With the introduction of holographic devices, holograms generated from CCT data can be projected into physical spaces, such as operating rooms, allowing operators to interact with them in real-time during both pre- and intraoperative phases, simulating the presence of tangible objects [167–169]. Compared to the traditional imaging methods, CCT-generated mixed reality holograms predicted the presence of loops, kinking, fibrosis and estimated the degree of procedural difficulty more accurately and consistently, as the preoperative insights correlated with a higher degree to the intraoperative findings. By offering an enhanced understanding of cardiovascular anatomy and potential complications, mixed reality holograms can also be a valuable tool for educational purposes [167]. 

## Conclusion

CCT has proven a valuable imaging modality in the evaluation of patients with a wide spectrum of arrhythmias that necessitate an electrophysiological intervention. It preoperatively provides important anatomical information that impacts the patient´s eligibility for the intervention and guides the electrophysiologist in the procedural planning and avoidance of injury of important anatomical structures. Furthermore, it identifies anatomical factors that confer worse outcomes and can predict arrhythmia recurrence following the procedure. The integration of CCT data with EAM systems provides real time guidance, leading to increased procedural efficacy. Moreover, CCT is particularly useful in the preoperative and postoperative assessment of LAAO, providing comparable, if not better insights compared to the reference standard TEE. The availability of CCT, simplicity in performance and widespread application across all procedural phases allows for more efficient, faster and safer interventions. Moreover, CCT has a prominent role in the patient follow-up as it constitutes a main imaging tool for diagnosis of complications and can be used in their management. Many of the novel and innovative imaging–based approaches that according to contemporary studies can further enhance the outcomes of the electrophysiological procedures are based on CCT, underlying its highly promising role and suggesting that further capabilities of CCT, remain to be explored.

## Data Availability

No datasets were generated or analysed during the current study.

## Abbreviations

2D: Two-dimensional
3D: Three-dimensional
3DCM: Three-dimensional computational modeling
AEF: Atrioesophageal fistula
AF: Atrial fibrillation
ARVC: Arrhythmogenic right ventricular cardiomyopathy
CCT: Cardiac computed tomography
CMR: Cardiac magnetic resonance
CRT: Cardiac resynchronization therapy
EAM: Electroanatomical mapping
HU: Hounsfield unit
IAS: Interatrial septum
LA: Left atrium
LAA: Left atrial appendage
LAAO: Left atrial appendage occlusion
LV: Left ventricle
NICM: Non-ischemic cardiomyopathy
PV: Pulmonary vein
PVS: Pulmonary vein stenosis
SBRT: Stereotactic body radiotherapy
TEE: Transesophageal echocardiography
TSP: Transseptal puncture
VT: Ventricular tachycardia
